# Biomechanical study of isolated radial head dislocation

**DOI:** 10.1186/s12891-017-1829-1

**Published:** 2017-11-21

**Authors:** Naoki Hayami, Shohei Omokawa, Akio Iida, Jirachart Kraisarin, Hisao Moritomo, Pasuk Mahakkanukrauh, Takamasa Shimizu, Kenji Kawamura, Yasuhito Tanaka

**Affiliations:** 1 Department of Orthopedic Surgery, Nara Medical University, 840 Shijo-cho, Kashihara City, Nara Prefecture Japan; 2 Department of Hand Surgery, Nara Medical University, 840 Shijo-cho, Kashihara City, Nara Prefecture Japan; 3 Department of Orthopedic Surgery, Faculty of Medicine, Chiang Mai University, Chiang Mai, 50200 Thailand; 4 Department of Physiotherapy, Osaka Yukioka College of Health Science, 41,1,1, Soujiji, Ibaraki City, Osaka Japan; 5 Department of Anatomy, Faculty of Medicine, Chiang Mai University, Chiang Mai, 50200 Thailand; 6 Excellence in Osteology Research and Training Center (ORTC), Chiang Mai University, Chiang Mai, 50200 Thailand

**Keywords:** Radial head dislocation, Biomechanical study, Annular ligament, Quadrate ligament, Interosseous membrane, Ligament reconstruction

## Abstract

**Background:**

Isolated radial head dislocation is a rare injury with an unclear pathomechanism, and the treatment is controversial. The purpose of the present study was to investigate the biomechanical contributions of the annular ligament, quadrate ligament, interosseous membrane, and annular ligament reconstructions to proximal radioulnar joint stability.

**Methods:**

Five fresh frozen cadaveric upper extremities were amputated above the elbow and solidly fixed on a customized jig. Radial head dislocation was reproduced by sequential sectioning of ligamentous structures and passive mobility testing. Radial head displacement during mobility testing was measured with an electromagnetic tracking device in three forearm rotation positions. The data were compared among different sectioning stages and between two types of simulated ligamentous reconstruction.

**Results:**

Lateral displacement of the radial head significantly increased in the neutral forearm rotation after annular ligament sectioning (46 ± 10%, *p* < 0.05). After quadrate ligament sectioning, we found significant posterior (67 ± 36%, p < 0.05) and lateral (74 ± 24%, *p* < 0.01) displacement in neutral forearm rotation and pronation. Significant radial head displacement was found in all directions and in all forearm positions after sequential sectioning of the proximal half of the interosseous membrane. Anatomical annular ligament reconstruction stabilized the proximal radioulnar joint except for anterior laxity in neutral forearm rotation (15 ± 6%, p < 0.05). The radial head with Bell Tawse procedure was significantly displaced in all directions.

**Conclusion:**

The direction of radial head instability varied depending on the degree of soft tissue sectioning and specific forearm rotation. Anterior radial head dislocation may involve more severe ligament damage than other types of dislocation. Anatomical annular ligament reconstruction provided multidirectional radial head stability.

## Background

Traumatic dislocation of the radial head is usually associated with fractures of the forearm; isolated dislocation is a rare injury [1]. Anterior radial head dislocation is the most common form, but its pathomechanism remains obscure. Although the annular ligament is the primary stabilizer of the proximal radioulnar joint [2, 3], the contributions of other stabilizing structures such as the quadrate ligament and interosseous membrane (IOM) are not completely understood. A biomechanical study investigating the relative contributions of the annular ligament and the IOM (proximal, central, and distal bands) to preventing radial head dislocation [4] found that no single structure provided a significantly different percentage of static stability. Although those authors demonstrated the importance of the central band of the IOM in providing dynamic stability during forearm rotation, few specimens showed radial head dislocation, and there was minimal displacement (2–3 mm) in their motion simulation experiment. Moreover, it remains unclear how the quadrate ligament contributes to radial head stability [5, 6].

When radial head dislocation is neglected at the time of injury, surgical intervention is frequently needed. Resection or open reduction of the radial head with either annular ligament reconstruction [7, 8] or ulnar corrective osteotomy is generally performed. One of the most popular techniques for ligament reconstruction is the Bell Tawse procedure, where a slip of the triceps tendon is passed around the radial neck and secures it through a drill hole in the ulna. However, there is no single preferred procedure.

The present study aimed to investigate the contributions of the annular ligament, quadrate ligament, and IOM to the stability of the proximal radioulnar joint in a simulated radial head dislocation model using cadaveric specimens. Furthermore, restoration of joint stability was evaluated following both anatomic and non-anatomic (Bell Tawse procedure) ligamentous reconstructions.

We hypothesized that isolated radial head dislocation would be reproduced not by sectioning of the annular ligament alone but would require additional sectioning of the quadrate ligament and IOM and that the direction and degree of radial head instability would vary depending on the severity of ligament sectioning and on forearm position. Determining the key stabilizer to prevent each direction of radial head dislocation and the optimal forearm position to ensure post-reduction stability would provide useful information in the management of patients with radial head dislocation.

## Methods

### (specimen preparation)

We used five fresh frozen cadaver upper extremities (from three males and two females; average age, 63 years). None of the specimens had any skeletal or articular pathology of the elbow joint or forearm bones. All specimens were amputated above the elbow, and the wrists were also disarticulated at room temperature before use and were kept moist by spraying with normal saline during the experiment. Skin, muscle, and tendons were removed; ligaments around the elbow joint and the IOM were preserved.

### (experimental setup)

**Fig. 1:**
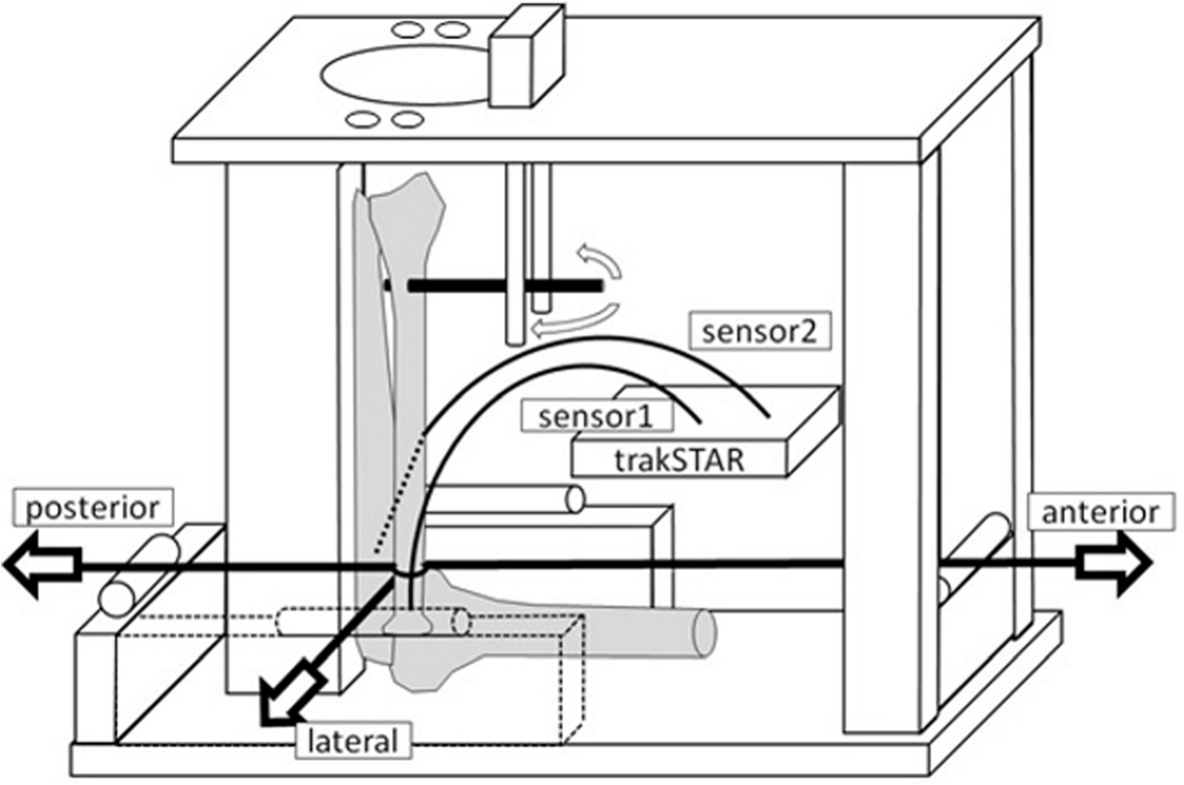
Experimental setup. The humerus and ulna were solidly fixed on a customized wooden jig with the elbow flexed at 90°. The humerus was set horizontally and ulna was set perpendicularly to the ground. The radius was kept in one of three forearm positions (full pronation, neutral rotation, or full supination) with a 2.0-mm K-wire inserted in the distal radius only to keep forearm rotation. The displacement of the radial head relative to the proximal ulna was measured by an electromagnetic tracking device (trak STAR™; Ascension Technology Corporation, Shelburne, VT, USA). Sensors were inserted into the radial head and the proximal ulna. A cord was positioned around the neck of the radial head to apply load for passive mobility testing

The humerus and ulna were solidly fixed on a customized wooden jig with the elbow flexed at 90°. The radius was kept in one of three forearm positions (full pronation, neutral rotation, or full supination) with a 2.0-mm K-wire inserted in the distal radius from radial aspect (Fig. 1). We regarded as neutral rotation when this K-wire and long axis of humerus became parallel. We used an electromagnetic tracking device (trakSTAR™; Ascension Technology Corporation, Shelburne, VT, USA) to measure the displacement of the radial head relative to the proximal ulna. Sensors were inserted into the radial head and the proximal ulna. A cord was positioned around the neck of the radial head to apply load for passive mobility testing.

### (passive mobility testing and data acquisition)

We performed passive mobility testing by loading the radial head with 20 N in the anterior, lateral, and posterior directions. A custom-made device prevented forearm rotation during passive mobility testing. We calculated the radial head displacement as a percentage of the radial head diameter by measuring position before and after loading the radial head in full pronation, neutral rotation, and full supination. The displacement ratio of the radial head was calculated from these data. These procedures were repeated at each sectioning stage and reconstruction stage as indicated below.

### (sectioning of radial head stabilizers)

**Fig. 2:**
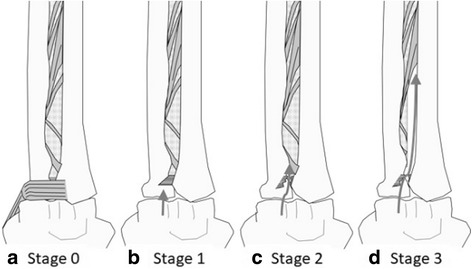
Sectioning stages of radial head stabilizers. **a** Stage 0: elbow with anterior joint capsule sectioned. **b** Stage 1: elbow with annular ligament sectioned. **c** Stage 2: elbow with annular and quadrate ligaments sectioned. **d** Stage 3: elbow with annular and quadrate ligaments and proximal half of the IOM sectioned

We simulated radial head instability with the ligaments intact and with sequential sectioning of the elbow ligaments and IOM (Fig. 2). Sectioning stages were defined as follows: stage 0, elbow with anterior joint capsule sectioned; stage 1, elbow with annular ligament sectioned; stage 2, elbow with annular and quadrate ligaments sectioned; stage 3, elbow with annular and quadrate ligaments and proximal half of the IOM sectioned.

### (annular ligament reconstruction)

After sectioning the ligaments and the IOM, we reconstructed the annular ligament with the following two procedures: stage R1, annular ligament reconstruction according to Bell Tawse procedure [9]; stage R2, anatomical annular ligament reconstruction with a triceps tendon slip passed around the radial neck through a bony tunnel created at the original ligamentous attachment sites on the ulna parallel to the proximal radioulnar joint and just distal to the radial notch. This is a modification of the procedure described by Itadera [10], in which a palmaris longus tendon graft is used. We used a distally based central one-third (4 mm in width) triceps tendon slip for each reconstruction and evaluated radial head instability in neutral forearm rotation.

### (statistical analysis)

Statistical analysis was performed with SPSS version 22 for Windows (SPSS Inc., Chicago, IL, USA). The radial head displacement ratio was analyzed with two-way repeated measures analysis of variance. After confirming the statistical significance of stages, forearm positions and their interaction, the data among sectioning stages were analyzed by Dunnett’s multiple comparison test to compare with stage 0. Displacement ratios were compared among different forearm positions with Tukey’s multiple comparison test. To evaluate the significance of annular ligament reconstruction, Tukey’s test was also used to compare the data among intact and two reconstruction stages.

## Results

### (incidence of radial head dislocation)

**Fig. 3:**
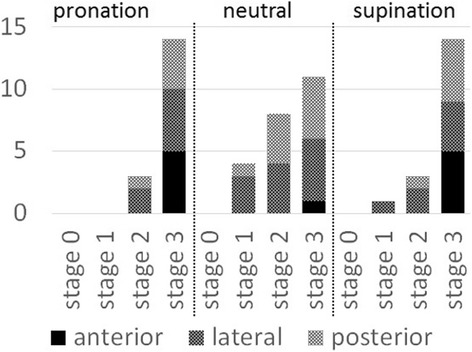
Incidence of radial head dislocation. According to ligament sectioning, the incidence of radial head dislocation increased. Radial head began to dislocate laterally after annular ligament sectioned. And next, posterior radial head dislocation was observed after quadrate ligament sectioned especially in neutral position. Anterior radial head dislocation was occurred after proximal half of IOM sectioned

We defined dislocation as more than 50% displacement  (Fig. 3). At stage 0, no radial head dislocation was observed. At stage 1, in neutral forearm rotation three radial heads (60%) dislocated laterally and one (20%) dislocated posteriorly. In supination, one radial head (20%) dislocated laterally. At stage 2, in neutral rotation there were four (80%) dislocations laterally and 4 (80%) posteriorly; during both supination and pronation, one radial head dislocated posteriorly and two dislocated laterally. No anterior dislocation was observed at stage 2, regardless of forearm position. At stage 3, four to five radial heads dislocated (80–100%) in each direction and in each forearm position except in the anterior direction in neutral forearm rotation (only one radial head, 20%).

### (displacement ratio of the radial head at each stage) 

#### Stage 0 (elbow with joint capsule sectioned)

The displacement ratio was less than 10% in all directions and in all forearm positions.

#### Stage 1 (elbow with annular ligament sectioned)

The radial head displacement ratio was significantly greater in the lateral direction in neutral rotation than at stage 0 (Table 1). The displacement ratio in the other tests was not significantly greater than at stage 0.

**Table 1:** Lateral radial head displacement ratio according to sectioning stage and forearm rotation (percentage; mean ± SD)

	pronation	neutral	supination
Stage 0	2 ± 1^a^	4 ± 1	3 ± 1
Stage 1	5 ± 3^a^	46 ± 10*	21 ± 25
Stage 2	41 ± 38*	74 ± 24**	42 ± 42
Stage 3	158 ± 22**	154 ± 30**	70 ± 42**^,b^

*: Indicates a value that is significantly different from that of stage 0 in the same column, with *p* < 0.05

**: Indicates a value that is significantly different from that of stage 0 in the same column, with p < 0.01

^a^Indicates a value that is significantly different from neutral rotation in the same row

^b^Indicates a value that is significantly different from the other positions in the same row

#### Stage 2 (elbow with annular and quadrate ligaments sectioned)

In neutral rotation, the posterior displacement ratio was significantly greater than at stage 0 as was the lateral displacement ratio (Table 2). In pronation, the lateral displacement ratio was significantly greater than at stage 0 (Table 1). In supination, there was no significant increase in displacement ratio compared with stage 0 in any direction. The anterior displacement ratio was not significantly greater than at other stages in any forearm rotation (Table 3).

**Table 2:** Posterior radial head displacement ratio according to sectioning stage and forearm rotation (percentage; mean ± SD)

	pronation	neutral	supination
Stage 0	2 ± 1	3 ± 1	2 ± 0
Stage 1	5 ± 2^a^	37 ± 17	7 ± 2
Stage 2	24 ± 37	67 ± 36*	34 ± 49
Stage 3	68 ± 33**^,a^	200 ± 40**	123 ± 56**^,a^

*: Indicates a value that is significantly different from that of stage 0 in the same column, with p < 0.05

**: Indicates a value that is significantly different from that of stage 0 in the same column, with p < 0.01

^a^Indicates a value that is significantly different from neutral rotation in the same row

**Table 3:** Anterior radial head displacement ratio according to sectioning stage and forearm rotation (percentage; mean ± SD)

	pronation	neutral	supination
Stage 0	3 ± 0	5 ± 2	4 ± 1
Stage 1	7 ± 3	8 ± 4	8 ± 4
Stage 2	12 ± 6	9 ± 3	12 ± 6
Stage 3	95 ± 31**	39 ± 14**^,a^	109 ± 35**

**: Indicates a value that is significantly different from that of stage 0 in the same column, with *p* < 0.01

^a^Indicates a value that is significantly different from the other positions in the same row

#### Stage 3 (elbow with annular and quadrate ligaments and proximal IOM sectioned)

The radial head was significantly displaced in all directions in all forearm rotations (Tables 1, 2 and 3). The anterior displacement ratio was only significantly greater at this stage than at stage 0 (Table 3).

#### Stage R1 (ligament reconstruction with bell Tawse procedure)

The anterior displacement ratio of the radial head averaged less than 20% in all forearm rotations. The displacement ratio tended to become larger in lateral and posterior directions. The radial head was significantly displaced in all directions (Table 4).

**Table 4:** Radial head displacement ratio in different directions according to reconstruction stage (percentage; mean ± SD)

	anterior	lateral	posterior
Stage 0	5 ± 2	4 ± 1	3 ± 1
Stage R1	15 ± 5**	70 ± 26**	118 ± 31**
Stage R2	15 ± 6*	20 ± 3	28 ± 26

*: Indicates a value that is significantly different from that of stage 0 in the same column, with p < 0.05

**: Indicates a value that is significantly different from that of stage 0 in the same column, with p < 0.01

#### Stage R2 (anatomical annular ligament reconstruction)

Anterior, lateral, and posterior displacement ratios averaged less than 15%, 20%, and 30%, respectively, in all forearm rotations. The radial head was stabilized multi-directionally except in the anterior direction in neutral forearm rotation (Table 4).

### (comparison of displacement ratio among three forearm positions) 

Comparing the anterior displacement, the displacement ratio was significantly smaller in neutral rotation than in pronation or supination at stage 3 (Table 3). Comparing the lateral displacement, the displacement ratio in neutral rotation was significantly larger than in pronation at stage 1. The displacement ratio in supination was significantly smaller than in the other positions at stage 3 (Table 1). Comparing the posterior displacement, the displacement ratio in pronation was significantly smaller than in neutral rotation at stages 1 and 3 (Table 2).

## Discussion

Numerous studies have examined distal radioulnar joint instability, but a paucity of information exists regarding the biomechanics of the proximal radioulnar joint. Although isolated radial head dislocation is a rare injury, understanding the stabilizing mechanism of the proximal radioulnar joint may help in the management of acute and chronic radial head dislocation. The current study revealed increasing displacement of the radial head during passive mobility testing with sequential sectioning of the proximal radioulnar joint-stabilizing structures from the proximal to distal direction. The radial head was dislocated in a lateral, posterior, and anterior direction depending on the degree of ligament sectioning; there were specific forearm positions in which the radial head was stabilized at each sectioning stage.

When the annular ligament was sectioned, lateral mobility of the radial head increased significantly in neutral forearm rotation; two of the five specimens dislocated laterally in this position. These results indicate that the annular ligament is a primary stabilizer in preventing lateral radial head dislocation.

After the additional sectioning of the quadrate ligament, significant posterior and lateral displacement of the radial head occurred. Posterior dislocation was found in two of the five specimens in neutral rotation. These results indicate that the quadrate ligament prevents posterior dislocation of the radial head. Spinner [5] and Wiley [3] described the functional anatomy of the quadrate ligament and reported that the ligament has a check–rein effect on the proximal radioulnar joint, tightening during pronation and supination and loosening in neutral forearm rotation. The current results support this check–rein effect of the quadrate ligament from a biomechanical point of view.

Significant anterior displacement occurred only after sequential sectioning of the proximal half of the IOM. These results indicate that the IOM is an important stabilizer in preventing anterior radial head dislocation, regardless of forearm position. In previous studies, the central band of the IOM was reported to be the stiffest stabilizing structure of the forearm [11, 12], contributing more to radial head stability than the annular ligament and proximal band of the IOM [4] during forearm pronation and supination. Thus, the presence of anterior radial head dislocation in clinical practice may suggest more severe soft tissue damage than that present with lateral and posterior dislocation.

There is no consensus on which position of forearm rotation is best for stabilization of the radial head. Several authors have recommended that the forearm be immobilized in supination rather than in neutral rotation or pronation, while others have recommend the neutral rotation after manual reduction of the radial head [8, 13–16]. The current results comparing stability among different forearm positions indicate that the ideal stabilizing position may differ depending on the direction of radial head dislocation. In patients with lateral radial head dislocation, supination may be recommended during post-reduction immobilization. In contrast, immobilization in pronation is recommended following closed reduction of posterior radial head dislocation [17]. The radius and ulna are crossed in pronation, and this bony contact may prevent posterior migration of the radial head. A neutral forearm rotation is recommended to stabilize the radial head following reduction of anterior dislocation with severe IOM injury, because the remaining distal IOM is tight in this position [18].

**Fig. 4:**
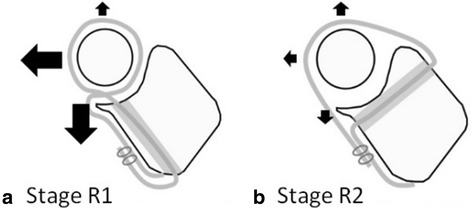
Schema of annular ligament reconstructions. **a** Stage R1: Bell Tawse procedure reduced anterior radial head dislocation; however, it was difficult to stabilize the radial head in the posterolateral direction with this technique because of the non-anatomical nature of the procedure. **b** Stage R2: Anatomical reconstruction stabilized the radial head in all directions

There are several surgical procedures available for the treatment of chronic radial head dislocation, including osteotomy of the ulna [19–22] with or without annular ligament reconstruction [16, 23–25]. Annular ligament reconstruction according to the Bell Tawse procedure is one popular technique, in which a slip of the triceps tendon is looped around the radial neck to stabilize the radial head. Although this procedure reduces anterior radial head dislocation, it is difficult to stabilize the radial head in the lateral and posterior directions with this technique because of the non-anatomical nature of the procedure (Fig. 4). Several clinical reports using this procedure for chronic radial head dislocation showed fair results with restricted forearm rotation and elbow flexion/extension [2, 26]. In the current study, the Bell Tawse procedure allowed posterolateral radial head instability, whereas anatomical reconstruction stabilized the radial head in all directions. Anatomical annular ligament reconstruction may provide multidirectional stability of the proximal radioulnar joint in patients with gross instability of the radial head. Thus, despite the rarity of the condition in clinical practice, the authors recommend performing anatomical annular ligament reconstruction rather than non-anatomical reconstruction procedures for patients with chronic radial head dislocation.

This study has several limitations. First, the soft tissue perhaps cannot tolerate repeated load and stretched during the experiment. To minimize this error, the load should not be too high, so 20 N of force was chosen. The same load was used previously in biomechanical studies of the wrist [27, 28]. Second, the sequence of ligament sectioning may differ from the clinical scenario in radial head dislocation. We modified the sequence of ligament sectioning from a previous biomechanical study [29], sectioning the stabilizing structures from the proximal to distal direction. A randomized or different sequence of ligamentous and IOM sectioning would provide additional information. Third, we did not assess the dynamic effects of the muscles around the radial head. Salama et al. reported anterior dislocation of the radial head after biceps contraction caused by electric shock [30]. The dynamic effect of the biceps muscle may increase anterior displacement.

## Conclusion

Isolated radial head dislocation is extremely rare. Nevertheless, the current study provided useful information related to the contributions of the annular ligament, the quadrate ligament, and the proximal half of IOM to radial head stability. Our findings may help with appropriate choice of post-reduction forearm position in acute injury and choice of reconstruction technique for chronic injury.

## Abbreviation

IOM: Interosseous membrane

## References

[1] Hamilton W, Parkes JC (1973). Isolated dislocation of the radial head without fracture of the ulna. Clin Orthop Relat Res.

[2] Hudson DA, De Beer JD (1986). Isolated traumatic dislocation of the radial head in children. J Bone Joint Surg Br..

[3] Wiley JJ, Pegington J, Horwich JP (1974). Traumatic dislocation of the radius at the elbow. J Bone Joint Surg Br..

[4] Anderson A, Werner FW, Tucci ER, Harley BJ (2015). Role of the interosseous membrane and annular ligament in stabilizing the proximal radial head. J Shoulder Elb Surg.

[5] Spinner M, Kaplan EB (1970). The quadrate ligament of the elbow--its relationship to the stability of the proximal radio-ulnar joint. Acta Orthop Scand.

[6] Tubbs RS, Shoja MM, Khaki AA, Lyerly M, Loukas M, O'neil JT, Salter EG, Oakes WJ (2006). The morphology and function of the quadrate ligament. Folia Morphol (Warsz).

[7] Oner FC, Diepstraten AF (1993). Treatment of chronic post-traumatic dislocation of the radial head in children. J Bone Joint Surg Br..

[8] Horii E, Nakamura R, Koh S, Inagaki H, Yajima H, Nakao E (2002). Surgical treatment for chronic radial head dislocation. J Bone Joint Surg Am.

[9] Bell Tawse AJ (1965). The treatment of malunited anterior Monteggia fractures in children. J Bone Joint Surg Br..

[10] Itadera E, Ueno K (2014). Recurrent anterior instability of the radial head: case report. J Hand Surg Am..

[11] Moritomo H, Noda K, Goto A, Murase T, Yoshikawa H, Sugamoto K (2009). Interosseous membrane of the forearm: length change of ligaments during forearm rotation. J Hand Surg Am..

[12] Werner FW, Taormina JL, Sutton LG, Harley BJ (2011). Structural properties of 6 forearm ligaments. J Hand Surg Am..

[13] Aversano F, Kepler CK, Blanco JS, Green DW (2011). Rare cause of block to reduction after radial head dislocation in children. J Orthop Trauma.

[14] Oka K, Murase T, Moritomo H, Sugamoto K, Yoshikawa H (2010). Morphologic evaluation of chronic radial head dislocation: three-dimensional and quantitative analyses. Clin Orthop Relat Res.

[15] Sasaki K, Miura H, Iwamoto Y (2006). Unusual anterior radial head dislocation associated with transposed biceps tendon: a case report. J Shoulder Elb Surg.

[16] Wang MN, Chang WN (2006). Chronic posttraumatic anterior dislocation of the radial head in children: thirteen cases treated by open reduction, ulnar osteotomy, and annular ligament reconstruction through a Boyd incision. J Orthop Trauma.

[17] Dhawan A, Hospodar PP (2002). Isolated posttraumatic posterior dislocation of the radial head in an adult. Am J Orthop (Belle Mead NJ).

[18] Malone PS, Cooley J, Morris J, Terenghi G, Lees VC (2015). The biomechanical and functional relationships of the proximal radioulnar joint, distal radioulnar joint, and interosseous ligament. J Hand Surg Eur Vol.

[19] Hasler CC, Von Laer L, Hell AK (2005). Open reduction, ulnar osteotomy and external fixation for chronic anterior dislocation of the head of the radius. J Bone Joint Surg Br..

[20] Exner GU (2001). Missed chronic anterior Monteggia lesion. Closed reduction by gradual lengthening and angulation of the ulna. J Bone Joint Surg Br..

[21] Bhaskar A (2009). Missed Monteggia fracture in children: is annular ligament reconstruction always required?. Indian J Orthop..

[22] Gyr BM, Stevens PM, Smith JT (2004). Chronic Monteggia fractures in children: outcome after treatment with the bell-Tawse procedure. J Pediatr Orthop B.

[23] Datta T, Chatterjee N, Pal AK, Das SK (2014). Evaluation of outcome of corrective ulnar osteotomy with bone grafting and annular ligament reconstruction in neglected monteggia fracture dislocation in children. J Clin Diagn Res.

[24] Tan L, Li YH, Sun DH, Zhu D, Ning SY (2015). Modified technique for correction of isolated radial head dislocation without apparent ulnar bowing: a retrospective case study. Int J Clin Exp Med.

[25] Garg P, Baid P, Sinha S, Ranjan R, Bandyopadhyay U, Mitra S (2011). Outcome of radial head preserving operations in missed Monteggia fracture in children. Indian J Orthop.

[26] Lloyd-Roberts GC, Bucknill TM (1977). Anterior dislocation of the radial head in children: aetiology, natural history and management. J Bone Joint Surg Br.

[27] Iida A, Omokawa S, Moritomo H, Omori S, Kataoka T, Aoki M, Wada T, Fujimiya M, Tanaka Y (2014). Effect of wrist position on distal radioulnar joint stability: a biomechanical study. J Orthop Res.

[28] Miyamura S, Shigi A, Kraisarin J, Omokawa S, Murase T, Yoshikawa H, Moritomo H (2017). Impact of Distal Ulnar Fracture Malunion on Distal Radioulnar Joint Instability: A Biomechanical Study of the Distal Interosseous Membrane Using a Cadaver Model. J Hand Surg Am.

[29] Weiss AP (1992). Hastings H 2nd.V. The anatomy of the proximal radioulnar joint. J Shoulder Elb Surg.

[30] Salama R, Wientroub S, Weissman SL (1977). Recurrent dislocation of the head of the radius. Clin Orthop Relat Res.

